# A Practical Treatise on the Domestic Management and Most Important Diseases of Advanced Life

**Published:** 1849-08

**Authors:** 


### BIBLIOGRAPHICAL NOTICES.

*A Practical Treatise on the Domestic Management and most important Diseases of advanced Life;* with an appendix, containing a series of cases illustrative of a new and successful mode of treating Lumbago, and other forms of Chronic Rheumatism, Sciatica and other neuralgic affections, and certain forms of Paralysis. By George E. Day, M. D., Fellow of the Royal College of Physicians, and Physician to the Western General Dispensary. Philadelphia: Lea & Blanchard; 1849, 12mo. pp. 226.

In addition to the above copious title page, we quote the following extract from the author's introduction, which will enable the reader to form an idea of the scope and character of the work:

"The want, that I personally experienced, of a standard work on this subject, led me, from the period I entered on the active duties of my profession, to note down for my own guidance, all the facts and observations bearing on the diseases of advanced life and their treatment, which my official connexion with large charitable institutions daily presented to me. I have likewise been in the habit of recording references to all the works, journals, &c., which in the ordinary course of reading I have found to contain any information on these points. The matter that has been thus gradually accumulating from my own experience, and from the records of other laborers in the same field, is now presented to the world in a very condensed form; but, in order to enable others to pursue with greater facility the same subject, or individual departments of it, I have appended to these remarks the bibliography which I have constructed. My great object has been to render this volume an essentially practical work, and with this view I have intentionally omitted any notice of the appearances presented after death from the diseases which I have described in the following pages. This omission will be supplied in a work which will shortly appear."

